# Sensitivity and Specificity of Natural Language Processing Systems for Identification of Hospitalized People Who Use Drugs

**DOI:** 10.1093/ofid/ofaf370

**Published:** 2025-06-23

**Authors:** Leah Benrubi, Taisuke Sato, Leo K Westgard, Kyle Zollo-Venecek, Brindet Socrates, Benjamin Sweigart, Jessica P Ridgway, Joji Suzuki, Yoelkys Morales, David Goodman-Meza, Alysse G Wurcel

**Affiliations:** School of Medicine, Tufts University, Boston, MA, USA; Tufts Medicine, Boston, MA, USA; College of Osteopathic Medicine, Kansas City University, Kansas City, MO, USA; Tufts Medicine, Boston, MA, USA; Tufts Medicine, Boston, MA, USA; Tufts Medicine, Boston, MA, USA; Department of Medicine, Boston Medical Center, Boston, MA, USA; Tufts Medicine, Boston, MA, USA; School of Medicine, University of Chicago, Chicago, IL, USA; Brigham and Women's Hospital, Boston, MA, USA; School of Medicine, Tufts University, Boston, MA, USA; Kirby Institute, University of New South Wales, Sydney, Australia; School of Medicine, Tufts University, Boston, MA, USA; Tufts Medicine, Boston, MA, USA; Department of Medicine, Boston Medical Center, Boston, MA, USA; School of Medicine, Boston University, Boston, MA, USA

**Keywords:** harm reduction, infectious diseases, injection drug use, natural language processing, people who use drugs

## Abstract

**Background:**

People who use drugs (PWUD) often lack access to optimal harm reduction and substance use disorder treatment tools. Tracking the epidemiology of acute care utilization by PWUD is crucial to improving systems of care. Chart reviews and *International Classification of Diseases* (*ICD*) codes are the most common systems of identifying hospitalizations of PWUD but are limited by high labor costs and inaccuracy. This study evaluates whether natural language processing (NLP) enhances the sensitivity and specificity of *ICD-10* codes in identifying hospitalizations of PWUD.

**Methods:**

We analyzed admissions at Tufts Medical Center between 2018 and 2023. Two NLP tools (Regular Expression and Open Health NLP Toolkit) were developed to identify PWUD and were compared with *ICD-10* algorithms. The NLP and *ICD-10* algorithms were applied to all admissions, and demographic and hospitalization-related data were extracted. The research team manually reviewed notes written during 790 hospitalizations of PWUD as the gold standard. We calculated sensitivity, specificity, and net reclassification indices.

**Results:**

*ICD-10* codes alone demonstrated low sensitivity (43%) but high specificity (99%). Adding NLP systems improved sensitivity up to 94%, though specificity decreased to 46%. Threshold adjustments (eg, notes flagged ≥50%) revealed a trade-off between sensitivity (47%) and specificity (96%). The most practical model—Regular Expression or *ICD-10* codes—resulted in a sensitivity of 74% and specificity of 87%.

**Conclusions:**

NLP is an innovative tool that can create functional, cost-effective, and accurate systems of identifying hospitalized PWUD. These findings support further development of NLP technologies to improve health care equity for PWUD.

## Teaser

This study investigates the use of natural language processing (NLP) in identifying the hospitalization encounters of people who use drugs. When compared with *ICD-10* codes alone, NLP improved sensitivity but decreased specificity. NLP is a promising avenue for improving health care for hospitalized people who use drugs.

## Graphical Abstract

**Graphical Abstract:**
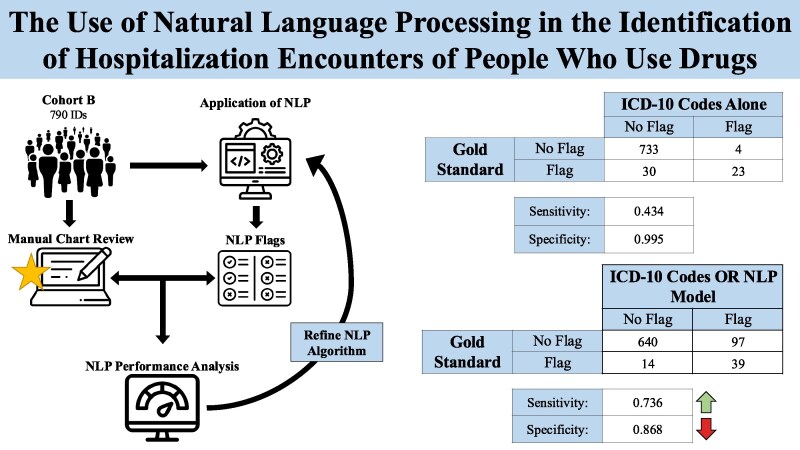

People who use drugs (PWUD) deserve the highest level of evidence-based health care. PWUD include people who snort, sniff, ingest, and inject drugs such as opioids, cocaine, and methamphetamines. Examples of evidence-based care for PWUD include prescribing medications for opioid use disorder, providing access to overdose-reversal medications, testing for infections, and offering infection prevention tools such as vaccines and preexposure prophylaxis [1, 2]. Acute care hospitalization utilization among PWUD has increased over the past decades, largely driven by sequalae of drug overdose and infections [3–6]. There are inadequate systems of support for hospital and posthospitalization care for PWUD [7, 8], leading to continued suboptimally treated substance use disorder (SUD) and infection.

Quality metrics for health care for the care of PWUD are largely absent—a stark contrast to the highly regulated and evaluated systems of care for diseases such as cardiovascular disease and diabetes. A barrier to developing quality metrics is the identification of PWUD. Most research on the epidemiology of health care utilization by PWUD has used *International Classification of Diseases* (*ICD*) codes [4, 9]. No *ICD-10* code exists for injection drug use (IDU) [10]; therefore, as surrogates for IDU, researchers use *ICD-10* codes for opioid and illicit drug use, diagnoses of substance abuse or dependence per the *Diagnostic and Statistical Manual of Mental Disorders*, and the presence of hepatitis C infection, among others [11–14]. A review of the literature with these methods demonstrates a range of sensitivities between 11% and 65%, specificities between 90% and 99%, and positive predictive values <70% [12, 15, 16]. It is likely that *ICD* codes alone are inaccurate in estimating the prevalence of IDU and incidence of serious injection-related infections [13, 17].

Natural language processing (NLP) is a field of computer science nested within artificial intelligence that has shown promise in improving the identification of PWUD [11, 18]. Broadly, NLP has been used to harness computer programming to recognize and flag key phrases of interest, such as “injects drugs” or “smoked crack,” that may appear as free text in notes written by clinicians. A benefit of NLP is that it can scan all sections of the record, including social history, to find excerpts of interest. NLP has been used for the identification of hospitalizations associated with opioid use disorder in previous studies [19–21] and occurrences of inpatient initiation of medications for opioid use disorder [11, 18].

There is an emerging literature supporting the use of NLP to support improved care systems, but overall the science of NLP is nascent. The goal of this research was to assess if NLP, when coupled with *ICD-10* codes, improved accurate identification of hospital encounters for PWUD.

## MATERIALS AND METHODS

### Setting and Design

This retrospective study analyzed electronic health records from all admissions to Tufts Medical Center (TMC) from 2018 to 2023 (henceforth, cohort A). TMC is a 415-bed academic tertiary hospital in Boston, Massachusetts. Located in Boston's Chinatown neighborhood, TMC serves a majority publicly insured population, with 38% and 22% of patients covered by Medicare and Medicaid, respectively, in 2022 [22].

### Development of NLP Systems of Identification

#### Selecting NLP Systems

Following the recommendation of a health care data analyst (K. Z.-V.), we selected 2 systems of identification to compare: Regular Expression (RegEx) and Open Health Natural Language Processing (OHNLP). Both systems were included because their contrasting strengths and weaknesses mean that they have different applications. RegEx is a computer science tool that identifies sequences of characters matching a specified pattern in text. While not a complete NLP system by itself, RegEx is often used as part of larger NLP systems and is useful because it allows for hyperspecificity, can be run in many programming languages, is familiar and cheap, and has high computational efficiency. RegEx has been used as a scientific method for identifying sensitive topics, including sexual behaviors and drug use, in electronic health records in previous research [23, 24].

In contrast, OHNLP is an open source biomedical-specific NLP system built on the Apache UIMA framework [25]. It works by organizing text processing into a modular pipeline of tools that detect clinical entities, relationships, and other structured information from unstructured clinical narratives. By combining rule-based methods with machine learning techniques, OHNLP enables researchers and clinicians to efficiently extract meaningful insights from complex health care data. This system of NLP analysis was first applied in 2010 by researchers at Mayo Clinic to clinical notes [26–28]. The OHNLP system provides much more guidance and flexibility than RegEx but is also correspondingly more complex, computationally expensive, and labor intensive. To date, the OHNLP toolkit has not been used to identify PWUD but has been used as a text annotation tool in several studies [29, 30].

#### Refining Text

**Figure 1.**
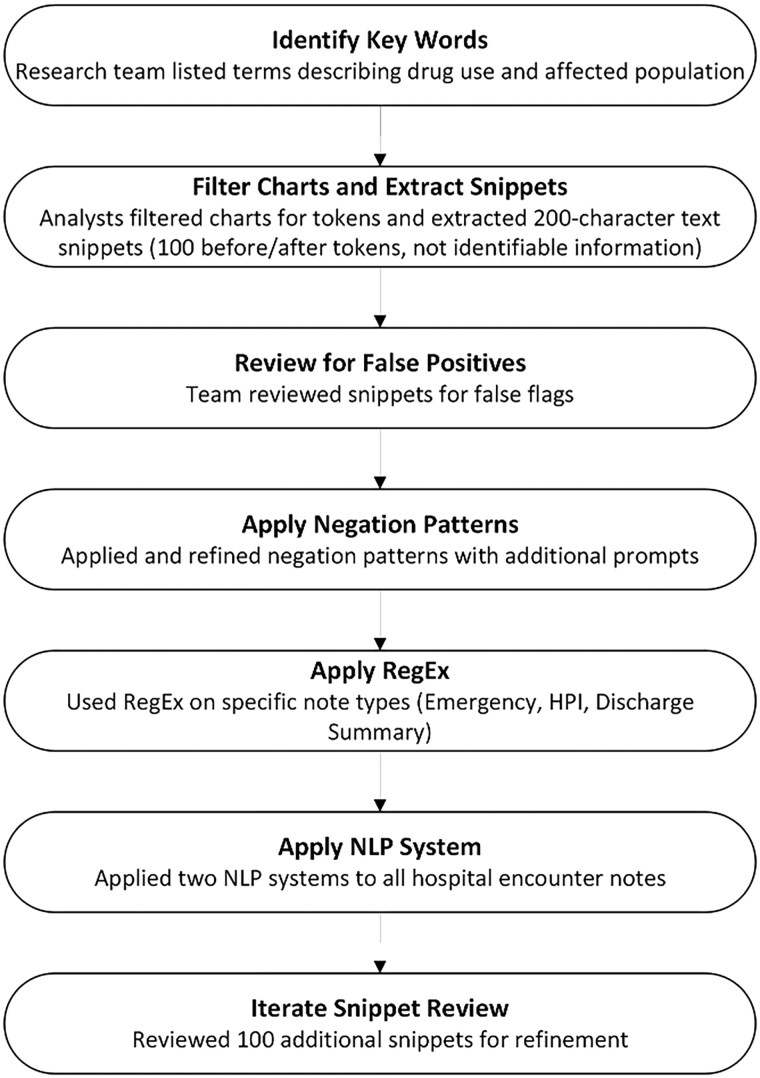
Flowchart describing the research team's process for defining terms, called *tokens*, to be applied by data analysts for NLP. This process required iterative review of terms and results before being applied to NLP models for use. Abbreviations: HPI, history of present illness; NLP, natural language processing.

Research team members (T. S. and A. G. W.) created a list of text and phrases commonly used to describe the action of drug use (eg, “injects drugs”), the PWUD (eg, “intravenous drug user”), and drugs (eg, heroin, cocaine) that the NLP tools could identify. Health care data analysts filtered charts based on the list of terms and provided examples of text from charts (“snippets”) that flagged positive for the query (n = 2702). The snippets included 100 characters before and after the matching word or phrase. No identifiable information was included in the snippet. Then, the research team reviewed the list of snippets to look for examples of false triggering (eg, “fentanyl gtt” means that the person was receiving fentanyl as a medication). A negation pattern was applied to eliminate instances with negated positive mentions (eg, “no IVDU”), and it was augmented with additional prompts for specification. This process occurred again with research team members reviewing 100 snippets (A. G. W. and B. Socrates). Figure 1 shows the process, and Supplementary Table 1 presents the final list of 40 words and phrases and the negations.

#### Applying NLP Systems

**Figure 2.**
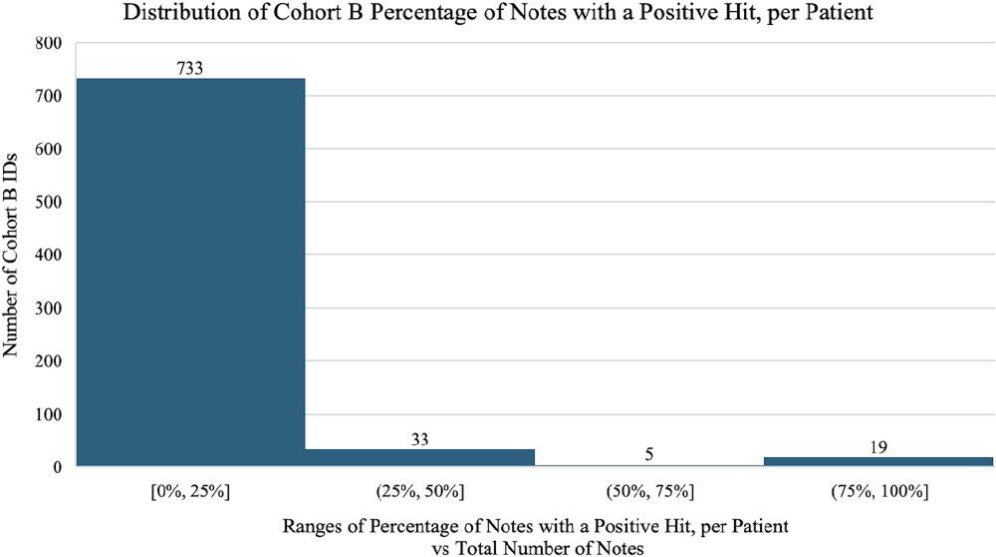
Distribution histogram describes the number of individuals of each percentage range by the percentage of notes per patient chart flagged positive by the Regular Expression model. This informed the cutoff points of 10%, 25%, and 50% that were used as models for which specificity and sensitivity were calculated.

First, we applied the NLP tools to all notes in all hospital encounters separately. Then, we applied the RegEx to specific note types (emergency note, “history of present illness” note, and discharge summary). The RegEx system reported the following data: the total number of notes linked to the hospital admission encounter, the total number of notes containing true RegEx terms (positive hits subtracting positive hits with negation), and RegEx terms that flagged positive. The outcome of NLP-flagged PWUD present per patient chart by RegEx was then characterized into 3 categories: present in at least 10%, 25%, and 50% of notes. These thresholds were determined by visualizing the distribution of all NLP-flagged PWUD present per patient chart as illustrated in Figure 2. The OHNLP system reported data as follows: the snippet identified, the note type, which term was identified, whether the system identified a history of drug use, and whether there was a possible negation. The OHNLP system was not applied to cohort A due to its high computational expense for a large data set; therefore, these results are not reported in Table 1.

**Table 1.** Demographic Information of Hospitalizations in Cohorts A and B, 2018–2023, Including Percentage of Charts Flagged Positive by *ICD-10* Code, OHNLP, or RegEx System

	Cohort A (N = 284 634)	Cohort B (n = 790)
Age, y		
Mean (SD)	48.3 (19.5)	43.9 (13.9)
Median (range)	48.0 (18.0–123)	44.0 (18.0–65.0)
Gender		
Female	137 533 (48.3)	469 (59.4)
Male	147 067 (51.7)	320 (40.5)
Missing	34 (0.0)	1 (0.1)
Race		
American Indian or Alaska Native	595 (0.2)	1 (0.1)
Asian	35 450 (12.5)	72 (9.1)
Asian Indian	1684 (0.6)	4 (0.5)
Black or African American	60 053 (21.1)	120 (15.2)
Missing	30 341 (10.7)	123 (15.6)
Native Hawaiian or other Pacific Islander	152 (0.1)	0 (0)
White	156 359 (54.9)	470 (59.5)
Ethnicity		
Hispanic or Latino	31 240 (11.0)	107 (13.5)
Missing	6121 (2.2)	36 (4.6)
Not Hispanic or Latino	247 273 (86.9)	647 (81.9)
Length of stay, d		
Mean (SD)	3.14 (30.9)	5.46 (10.3)
Median (range)	0 (0–1530)	3.00 (0–143)
Patient history of *ICD-10* codes associated with drug use		
Yes	24 047 (8.4)	42 (5.3)
No	260 587 (91.6)	748 (94.7)
Visit has *ICD-10* codes associated with drug use		
Yes	22 885 (8.0)	27 (3.4)
No	261 749 (92.0)	763 (96.6)
Patient history of and visit involving *ICD-10* codes associated with drug use		
Yes	22 885 (8.0)	15 (1.9)
No	261 749 (92.0)	775 (98.1)
OHNLP hit		
Yes	…	177 (22.4)
No	…	613 (77.6)
RegEx hit		
Yes	157 193 (55.2)	153 (19.4)
No	127 441 (44.8)	637 (80.6)
Notes with a RegEx hit		
≥10%	152 927 (53.7)	123 (15.6)
<10%	131 707 (46.3)	667 (84.4)
Notes with a RegEx Hit		
≥25%	172 593 (60.6)	68 (8.6)
<25%	112 041 (39.4)	722 (91.4)
Notes with a RegEx Hit		
≥50%	193 924 (68.1)	29 (3.7)
<50%	90 710 (31.9)	761 (96.3)

Data are presented as No. (%) unless noted otherwise. Cohort A, all admissions to Tufts Medical Center from 2018 to 2023; Cohort B, subset of encounters from Cohort A chosen at random.

Abbreviations: *ICD-10*, *International Statistical Classification of Diseases, Tenth Revision*; OHNLP, Open Health Natural Language Processing; RegEx, Regular Expression.

#### Application of Indicators of PWUD to Cohort

Following prior work by the research team and review of the literature, the research team compiled a list of *ICD-10* codes used previously for SUD identification [11, 14, 18, 31]. There were 2 systems of pulling *ICD-10* codes: the top 5 *ICD-10* codes linked to the hospital encounter and all *ICD-10* codes linked to the patients’ charts (Supplementary Table 2).

### Statistical Analysis

#### Sample Size Calculations

According to previous work, PWUD represented an estimated 10% of admissions to TMC [32]. A sample size of 790 patients achieves 80% power to detect a difference of 0.15 in the sensitivity between the search methods, assuming that the prevalence of PWUD hospitalizations is 0.10 and the proportion of discordant results among PWUD is 0.22. This procedure uses a 2-sided McNemar test with a significance level of .05.

#### Gold Standard

The gold standard for identification of health care encounters for PWUD is chart review [33]. A standard operating procedure developed by the research team (Y. M. and T. S.) guided the process of chart review (supplementary document). The procedure included examining (1) the emergency department documentation, (2) the admission or “history of present illness” note, and (3) the discharge summary related to the encounter. Two researchers (T. S. and Y. M.) independently chart reviewed a subset of encounters from cohort A chosen at random totaling 790 encounters, referred to as cohort B, and then confirmed intercoder reliability by comparing their initial coding results. If there was disagreement over the results, a third reviewer (A. G. W.) provided a tiebreaker.

#### Calculations of Sensitivity, Specificity, F Statistic, and Net Classification Index

Sensitivity, specificity, and F1 scores for drug use and non–drug use were calculated to evaluate models. A population-weighted net reclassification index (wNRI) was also utilized to separately compare the use of *ICD-10* codes associated with drug use from patients’ visits for drug use identification with the NLP models. The formula for the index is wNRI *=* ρNRI_e_ + (1 – ρ)NRI_ne_. In this formula, event net reclassification index (NRI_e_) is *P*(up|drug use) – *P*(down|drug use), and the nonevent net reclassification index (NRI_ne_) is *P*(down|no drug use) – *P*(up|no drug use), where *up* is an increased likelihood of identifying drug use and *down* is a decreased likelihood of identifying drug use. ρ is the prevalence of drug use in the study population. In other words, the NRI_e_ and NRI_ne_ compare the models’ net relative effectiveness in identifying PWUD and people who do not use drugs, respectively. NRI_e_ and NRI_ne_ are reported. All calculations were conducted in R version 4.4.1.

### Ethics Approval

This study received approval from the Tufts Health Sciences institutional review board.

### Patient Consent Statement

This study did not include factors necessitating patient consent.

## RESULTS

The characteristics of all admissions (N = 284 634, cohort A) and the subcohort for chart review (n = 790, cohort B) are described in Table 1. The calculated sensitivity, specificity, and F1 scores for the various models of NLP algorithms and *ICD-10* codes are shown in Table 2. Most models showed a high degree of specificity. In contrast, there was variability in the sensitivity of the models. The models that utilized the RegEx percentage categories and *ICD-10* codes were tested to verify whether a particular threshold performed more accurately than another. As the percentage threshold increased, the sensitivity of the models decreased while their specificity increased. For visits where a model predicted drug use, between 0% (*ICD-10* and OHNLP / *ICD-10* and RegEx) and 91% (OHNLP only / *ICD-10* or OHNLP) of predictions were correct. Based on F1 scores, the *ICD-10* and (RegEx or OHNLP) model performed the best (F1 score for identifying drug use, 0.568; F1 score for identifying non–drug use, 0.979).

**Table 2.** Calculated Sensitivity and Specificity of Various Testing Measures

	Measure^a^		F1 Score	
Category	Sensitivity	Specificity	Drug Use	Non–drug use
*ICD-10* only	0.434	0.995	0.597	0.979
RegEx only	0.604	0.836	0.315	0.899
OHNLP only	0.906	0.537	0.317	0.885
*ICD-10* or RegEx	0.641	0.753	0.389	0.903
*ICD-10* and RegEx	0.000	0.982	0.406	0.975
*ICD-10* or OHNLP	0.906	0.488	0.343	0.886
*ICD-10* and OHNLP	0.000	0.948	0.535	0.978
*ICD-10* or RegEx or OHNLP	0.943	0.465	0.282	0.813
*ICD-10* and (RegEx or OHNLP)	0.434	0.996	0.568	0.979
*ICD-10* or RegEx 10% threshold	0.736	0.868	0.419	0.923
*ICD-10* and RegEx 10% threshold	0.245	0.999	0.406	0.975
*ICD-10* or RegEx 25% threshold	0.623	0.931	0.493	0.953
*ICD-10* and RegEx 25% threshold	0.208	1.000	0.361	0.974
*ICD-10* or RegEx 50% threshold	0.472	0.965	0.495	0.966
*ICD-10* and RegEx 50% threshold	0.094	1.000	0.182	0.970

Abbreviations: *ICD-10*, *International Statistical Classification of Diseases, Tenth Revision*; OHNLP, Open Health Natural Language Processing; RegEx, Regular Expression.

^a^The gold standard was based on manual chart review and was considered positive if at least 2 of the 3 chart types (emergency, admission, and discharge notes) had language that characterized individuals as people who use drugs.

The calculated wNRIs, NRI_e_, and NRI_ne_ are reported in Table 3. Whereas the sensitivities and specificities shown in Table 2 show the accuracy of the models’ predictions given that they predicted drug use or non–drug use, these metrics compare models’ accuracy given that there was or was not drug use. The NRIs reveal that, due to their increased sensitivity, RegEx and OHNLP were better at identifying drug use relative to *ICD-10* codes alone. This also meant that they were less accurate in identifying non–drug use in more patients. Similarly, models requiring either an *ICD-10* code associated with drug use or a positive NLP flag were worse at predicting no drug use due to their increased sensitivity. Contrasting improvements in NRI_e_, particularly with the *ICD-10* or RegEx model, show that these same pairings helped identify a substantial number of visits involving drug use that were not identified through use of *ICD-10* alone. Requiring an *ICD-10* code associated with drug use and positive flag by 1 or both NLP models had a limited effect on net performance relative to the use of *ICD-10* codes alone. Marginal net improvements in non–drug use prediction were mildly offset by a corresponding decrease in drug use prediction performance.

**Table 3.** Net Reclassification Indices

Model^a^	wNRI	NRI_e_	NRI_ne_
*ICD-10* vs RegEx	−0.137	0.180	−0.158
*ICD-10* vs OHNLP	−0.157	0.260	−0.185
*ICD-10* vs *ICD-10* or RegEx	−0.128	0.380	−0.162
*ICD-10* vs *ICD-10* and RegEx	−0.009	−0.200	0.004
*ICD-10* vs *ICD-10* or OHNLP	−0.154	0.340	−0.188
*ICD-10* vs *ICD-10* and OHNLP	−0.003	−0.080	0.003
*ICD-10* vs *ICD-10* or RegEx or OHNLP	−0.257	0.460	−0.305
*ICD-10* vs *ICD-10* and (RegEx or OHNLP)	−0.001	−0.040	0.001
*ICD-10* vs *ICD-10* or RegEx 10% threshold	−0.097	0.020	−0.118
*ICD-10* vs *ICD-10* and RegEx 10% threshold	−0.009	−0.013	0.004
*ICD-10* vs *ICD-10* or RegEx 25% threshold	−0.047	0.013	−0.059
*ICD-10* vs *ICD-10* and RegEx 25% threshold	−0.010	−0.015	0.005
*ICD-10* vs *ICD-10* or RegEx 50% threshold	−0.025	0.003	−0.028
*ICD-10* vs *ICD-10* and RegEx 50% threshold	−0.018	−0.023	0.005

Abbreviations: *ICD-10*, *International Statistical Classification of Diseases, Tenth Revision*; NRI_e_, event net reclassification index; NRI_ne_, nonevent net reclassification index; OHNLP, Open Health Natural Language Processing; RegEx, Regular Expression; wNRI, population-weighted net reclassification index.

^a^All models are compared against the use of *ICD-10* codes from patient visits only. Formula described in Methods.

## DISCUSSION

Our study highlights the potential of NLP for identifying PWUD. Both NLP application systems demonstrated an improved ability to identify drug use over *ICD-10* codes alone, but this came at the cost of decreased specificity. However, a lower threshold for RegEx may offer reasonable sensitivity and specificity of 74% and 87%, respectively. These findings underscore the benefits and challenges inherent in leveraging NLP for the analysis of free-text variables. This project is also a clear testament to how a team of infectious diseases researchers, supported by informatics specialists, could develop and measure the impact of NLP on identifying hospitalized PWUD.

This work builds on earlier studies in which NLP systems were deployed to identify PWUD. In a 2022 study within Veterans Affairs, NLP was used to identify people who inject drugs among veterans diagnosed with *Staphylococcus aureus* bacteremia [18]. The findings included high sensitivity and specificity by using random forest modeling with RegEx filters for negation in comparison with *ICD-*based algorithms. Yet, this was limited to a majority male population and restricted to patients with a known risk factor of IDU. In comparison with this earlier work, our data are novel in that we attempted to use NLP on a generalized population without restrictions for disease type and focused on reducing the computational costs to increase feasibility for further hospital application.

Hospitalization represents a critical touchpoint for delivering evidence-based care to PWUD [34], as established by standards including guideline statements by the Society of Hospital Medicine published in September 2022 [35]. However, unlike conditions such as cardiovascular disease, where care delivery is standardized and systematically tracked [36], the management of PWUD shows significant variation across institutions and providers, including our home institution [32, 37]. This lack of standardization stems from the absence of reliable systems to identify patients at risk due to the low specificity of *ICD* codes for SUD [14, 33]. NLP offers a vital solution by including free-text variables, enabling the accurate identification of PWUD. NLP has already been leveraged by gastroenterologists [38], cardiologists [39], and pulmonologists [40]. Hospitals, clinicians, and people admitted to the hospital unrelated to SUD all stand to benefit from technological investment in improving health care to PWUD.

Harnessing the tools of NLP can improve the way that we benchmark quality care for PWUD. Currently, no state or federal systems are in place to track health care compliance with evidence-based interventions that support PWUD. NLP can be applied to tracking the epidemiology of serious injection-related infection and overdose in this population, monitoring hospital utilization, and ultimately informing the appropriate allocation of resources to support evidence-based treatment for PWUD. Several disease processes, such as cardiac surgery outcomes, are reported to the public and used as markers of quality care. A similarly robust system of tracking PWUD-related hospitalizations could facilitate comparable levels of care. Benchmarking across hospitals for patient-directed discharge rates in PWUD could identify centers that need support building up evidence-based SUD treatment protocols. It is worth noting that the algorithm built to identify encounters with PWUD should be tailored to fit the context. For example, if the algorithm is meant to identify in real time newly hospitalized PWUD for enrollment into a research study, a highly sensitive but not specific system could be a first pass, followed by human review. If the PWUD system of identification is, conversely, built to use only administrative-level data for population health-level assessments, it could be better to have a highly specific and but less sensitive system. We also need to state that systems using NLP should be codeveloped with people from the community who have lived experience of hospitalization related to SUD. Some of our previous work on an editorial piece about the benefits and risks of *ICD-10* code specifically for IDU highlighted the potential for harm as more specific administrative codes are created to capture substance use [10]. Additionally, the medical system has caused harm to PWUD, and this needs to be recognized proactively in the development of technological tools that may have unintended consequences of enhanced systems of surveillance [41]. Although there have been efforts to protect privacy, health care documentation in electronic medical records has caused consequences for PWUD [42].

Several limitations exist in this work. First, our analysis is dependent on the documentation from patient encounters, which may have missing information about drug usage by the clinician or from the patient due to concerns about stigmatization or legality. Therefore, the true number of patient encounters involving PWUD may be underrepresented in our data. Another significant limitation in our data analysis was the computational expense when the established OHNLP methodology was used on larger datasets. We switched to a lighter system that allowed more flexibility and ability to apply to more charts, although this system struggled with handling negations and linguistic complexity. Therefore, we could not achieve acceptable sensitivity and specificity that leveraged the assets of either system with adequate ease of use. We envision the use of large language models (LLMs) to carry out the aims set forth by this article. LLMs have shown promise in free text–based medical research due to their ability to manipulate unstructured data and draw from complex linguistic patterns, without the need for explicit rule generation by researchers [43, 44]. The work with LLM will need to have heightened scrutiny around ethics and data protection [45]. Additionally, we did not perform an error analysis to evaluate the RegEx or OHNLP phrases that returned as false positives or negatives.

Our data should be used as a launchpad for future evaluations of machine learning in the field of improving care to PWUD. By leveraging NLP, health care systems can ensure that PWUD receive equitable, evidence-based care, and they can facilitate the tracking of quality metrics essential for improving outcomes in this vulnerable population.

## Supplementary Material

ofaf370_Supplementary_Data

## Supplementary Data

Supplementary materials are available at *Open Forum Infectious Diseases* online. Consisting of data provided by the authors to benefit the reader, the posted materials are not copyedited and are the sole responsibility of the authors, so questions or comments should be addressed to the corresponding author.

## Notes

***Acknowledgments.*** We thank Clark Evans for his assistance on data collection.

***Author contributions.*** L. B.: validation, investigation, writing–original draft, writing–review and editing. T. S.: validation, investigation, visualization. L. K. W.: formal analysis, writing–original draft. K. Z.-V.: investigation, data curation. B. Socrates: formal analysis, writing–original draft, project administration. B. Sweigart: methodology. J. P. R.: conceptualization. J. S.: conceptualization. Y. M.: validation, investigation. D. G.-M.: conceptualization, methodology. A. G. W.: conceptualization, methodology, supervision, funding acquisition, writing–review and editing.

***Financial support.*** This work was supported by the S-GATS (Small Grants to Advance Translational Science) grant through Tufts CTSI (Clinical and Translational Science Institute) (UM1TR004398).
