# Supplementary material for: Sensitivity and Specificity of Natural Language Processing Systems for Identification of Hospitalized People Who Use Drugs
Source: Open Forum Infect Dis. 2025 Jun 23;12(7):ofaf370. doi: 10.1093/ofid/ofaf370 (PMC12264426; doi:10.1093/ofid/ofaf370)
Supplement: ofaf370_Supplementary_Data [file ofaf370_supplementary_data.docx]

**Supplementary Table 1**

*A: List of words and phrases generated and reviewed by research team for use in Natural Language Processing*

| **RegEx NLP Terms** |
| --- |
| IVDA |
| IVDU |
| IDU |
| injects drugs |
| injects heroin |
| injects fentanyl |
| injection drug use |
| heroin |
| fentanyl |
| methadone |
| suboxone |
| injection drug use |
| intravenous drug use |
| iv user |
| iv drug |
| drug abuse |
| OUD |
| SUD |
| opioid abuse |
| illicit drugs |
| substance use disorder |
| opioid use disorder |
| opioid overdose |
| methamphetamine |
| drug user |
| benzodiazepine abuse |
| overdose |
| amphetamine |
| PWUD |
| psychoactive substance use disorder |
| drug misuse |
| polysubstance |
| narcotic |
| addiction |
| addicted |
| addict |
| MOUD |
| cocaine |
| substance user |
| drug user |

*B: List of negations generated and reviewed by research team for use in Natural Language Processing*

| **Negations Terms IN FRONT of Matched Term** | **Negation Terms FOLLOWING Matched Term** | **Negation Filter** | **Negation Filter: Fentanyl ONLY** |
| --- | --- | --- | --- |
| No | Never | narcotic infus | sedation |
| No Recreational | None | narcotic medica | Adminst |
| No hx of | Denies | narcotic pain medica | mcg |
| No illicit | Defer | dextroamphetamine amphetamine | mg |
| Denies | None | home meds amphetamine | dosage |
| No History | Denies any recreational | nkda meds | unit |
|  | Denies other | nyquil | given |
|  | Denies any recreational | gabapentin | IV |
|  | Denies recreational | use assessment history |  |
|  | None | colace or senna |  |
|  | assessment history | narcotic refills |  |
|  | topics | substance use topics |  |
|  | medicat | drug use of any k |  |
|  |  | operate heavy machinery |  |
|  |  | there is a risk of addiction with narcotic |  |
|  |  | cyproheptadine |  |
|  |  | tylenol |  |
|  |  | over the counter |  |
|  |  | prescribed med |  |

**Supplementary Table 2: ICD-10 Codes used for Identifying People Who Use Drugs**

F11.1, Opioid abuse

F11.10, Opioid abuse, uncomplicated

F11.10, Opioid abuse, uncomplicated

F11.11, Opioid abuse, in remission

F11.12, Opioid abuse with intoxication

F11.120, Opioid abuse with intoxication, uncomplicated

F11.121, Opioid abuse with intoxication delirium

F11.122, Opioid abuse with intoxication with perceptual disturbance

F11.129, Opioid abuse with intoxication, unspecified

F11.13, Opioid abuse with withdrawal

F11.14, Opioid abuse with opioid-induced mood disorder

F11.15, Opioid abuse with opioid-induced psychotic disorder

F11.150, Opioid abuse with opioid-induced psychotic disorder with delusions

F11.151, Opioid abuse with opioid-induced psychotic disorder with hallucinations

F11.159, Opioid abuse with opioid-induced psychotic disorder, unspecified

F11.18, Opioid abuse with other opioid-induced disorder

F11.181, Opioid abuse with opioid-induced sexual dysfunction

F11.182, Opioid abuse with opioid-induced sleep disorder

F11.188, Opioid abuse with other opioid-induced disorder

F11.19, Opioid abuse with unspecified opioid-induced disorder

F11.2, Opioid dependence

F11.20, Opioid dependence, uncomplicated

F11.21, Opioid dependence, in remission

F11.21, Opioid dependence, in remission

F11.22, Opioid dependence with intoxication

F11.220, Opioid dependence with intoxication, uncomplicated

F11.221, Opioid dependence with intoxication delirium

F11.222, Opioid dependence with intoxication with perceptual disturbance

F11.229, Opioid dependence with intoxication, unspecified

F11.23, Opioid dependence with withdrawal

F11.24, Opioid dependence with opioid-induced mood disorder

F11.25, Opioid dependence with opioid-induced psychotic disorder

F11.250, Opioid dependence with opioid-induced psychotic disorder with delusions

F11.251, Opioid dependence with opioid-induced psychotic disorder with hallucinations

F11.259, Opioid dependence with opioid-induced psychotic disorder, unspecified

F11.28, Opioid dependence with other opioid-induced disorder

F11.281, Opioid dependence with opioid-induced sexual dysfunction

F11.282, Opioid dependence with opioid-induced sleep disorder

F11.288, Opioid dependence with other opioid-induced disorder

F11.29, Opioid dependence with unspecified opioid-induced disorder

F11.90, Opioid use, unspecified, uncomplicated

F11.92, Opioid use, unspecified with intoxication

F11.93, Opioid use, unspecified with withdrawal

F11.94, Opioid use, unspecified with opioid-induced mood disorder

F11.95, Opioid use, unspecified with opioid-induced psychotic disorder

F11.98, Opioid use, unspecified with other specified opioid-induced disorder

F11.99, Opioid use, unspecified with unspecified opioid-induced disorderF14.1, Cocaine abuse

F14.10, Cocaine abuse, uncomplicated

F14.11, Cocaine abuse, in remission

F14.12, Cocaine abuse with intoxication

F14.120, Cocaine abuse with intoxication, uncomplicated

F14.121, Cocaine abuse with intoxication with delirium

F14.122, Cocaine abuse with intoxication with perceptual disturbance

F14.129, Cocaine abuse with intoxication, unspecified

F14.13, Cocaine abuse, unspecified with withdrawal)

F14.14, Cocaine abuse with cocaine-induced mood disorder

F14.15, Cocaine abuse with cocaine-induced psychotic disorder

F14.150, Cocaine abuse with cocaine-induced psychotic disorder with delusions

F14.151, Cocaine abuse with cocaine-induced psychotic disorder with hallucinations

F14.159, Cocaine abuse with cocaine-induced psychotic disorder, unspecified

F14.18, Cocaine abuse with other cocaine-induced disorder

F14.180, Cocaine abuse with cocaine-induced anxiety disorder

F14.181, Cocaine abuse with cocaine-induced sexual dysfunction

F14.182, Cocaine abuse with cocaine-induced sleep disorder

F14.188, Cocaine abuse with other cocaine-induced disorder

F14.19, Cocaine abuse with unspecified cocaine-induced disorder

F14.2, Cocaine dependence

F14.20, Cocaine dependence, uncomplicated

F14.21, Cocaine dependence, in remission

F14.22, Cocaine dependence with intoxication

F14.220, Cocaine dependence with intoxication, uncomplicated

F14.221, Cocaine dependence with intoxication delirium

F14.222, Cocaine dependence with intoxication with perceptual disturbance

F14.229, Cocaine dependence with intoxication, unspecified

F14.23, Cocaine dependence with withdrawal

F14.24, Cocaine dependence with cocaine-induced mood disorder

F14.25, Cocaine dependence with cocaine-induced psychotic disorder

F14.250, Cocaine dependence with cocaine-induced psychotic disorder with delusions

F14.251, Cocaine dependence with cocaine-induced psychotic disorder with hallucinations

F14.259, Cocaine dependence with cocaine-induced psychotic disorder, unspecified

F14.28, Cocaine dependence with other cocaine-induced disorder

F14.280, Cocaine dependence with cocaine-induced anxiety disorder

F14.281, Cocaine dependence with cocaine-induced sexual dysfunction

F14.281, Cocaine dependence with cocaine-induced sexual dysfunction

F14.282, Cocaine dependence with cocaine-induced sleep disorder

F14.288, Cocaine dependence with other cocaine-induced disorder

F14.29, Cocaine dependence with unspecified cocaine-induced disorder

F14.92, Cocaine use, unspecified with intoxication

F14.920, Cocaine use, unspecified with intoxication, uncomplicated

F14.921, Cocaine use, unspecified with intoxication delirium

F14.922, Cocaine use, unspecified with intoxication with perceptual disturbance

F14.929, Cocaine use, unspecified with intoxication, unspecified

F15.1, Other stimulant abuse

F15.10, Other stimulant abuse, uncomplicated

F15.11, Other stimulant abuse, in remission

F15.12, Other stimulant abuse with intoxication

F15.120, Other stimulant abuse with intoxication, uncomplicated

F15.121, Other stimulant abuse with intoxication delirium

F15.122, Other stimulant abuse with intoxication with perceptual disturbance

F15.129, Other stimulant abuse with intoxication, unspecified

F15.13, Other stimulant abuse with withdrawal

F15.14, Other stimulant abuse with stimulant-induced mood disorder

F15.15, Other stimulant abuse with stimulant-induced psychotic disorder

F15.150, Other stimulant abuse with stimulant-induced psychotic disorder with delusions

F15.151, Other stimulant abuse with stimulant-induced psychotic disorder with hallucinations

F15.159, Other stimulant abuse with stimulant-induced psychotic disorder, unspecified

F15.18, Other stimulant abuse with other stimulant-induced disorder

F15.180, Other stimulant abuse with stimulant-induced anxiety disorder

F15.181, Other stimulant abuse with stimulant-induced sexual dysfunction

F15.182, Other stimulant abuse with stimulant-induced sleep disorder

F15.188, Other stimulant abuse with other stimulant-induced disorder

F15.19, Other stimulant abuse with unspecified stimulant-induced disorder

F15.2, Other stimulant dependence

F15.20, Other stimulant dependence, uncomplicated

F15.21, Other stimulant dependence, in remission

F15.22, Other stimulant dependence with intoxication

F15.22, Other stimulant dependence with intoxication

F15.220, Other stimulant dependence with intoxication, uncomplicated

F15.220, Other stimulant dependence with intoxication, uncomplicated

F15.221, Other stimulant dependence with intoxication delirium

F15.222, Other stimulant dependence with intoxication with perceptual disturbance

F15.229, Other stimulant dependence with intoxication, unspecified

F15.23, Other stimulant dependence with withdrawal

F15.24, Other stimulant dependence with stimulant-induced mood disorder

F15.25, Other stimulant dependence with stimulant-induced psychotic disorder

F15.250, Other stimulant dependence with stimulant-induced psychotic disorder with delusions

F15.251, Other stimulant dependence with stimulant-induced psychotic disorder with hallucinations

F15.259, Other stimulant dependence with stimulant-induced psychotic disorder, unspecified

F15.28, Other stimulant dependence with other stimulant-induced disorder

F15.280, Other stimulant dependence with stimulant-induced anxiety disorder

F15.281, Other stimulant dependence with stimulant-induced sexual dysfunction

F15.282, Other stimulant dependence with stimulant-induced sleep disorder

F15.288, Other stimulant dependence with other stimulant-induced disorder

F15.29, Other stimulant dependence with unspecified stimulant-induced disorder

R78.2, Finding of cocaine in blood

T40.0, Poisoning by, adverse effect of and underdosing of opium

T40.0X1, Poisoning by opium, accidental (unintentional)

T40.0X1A, Poisoning by opium, accidental (unintentional initial encounter

T40.0X1D, Poisoning by opium, accidental (unintentional subsequent encounter

T40.0X1D, Poisoning by opium, accidental (unintentional subsequent encounter

T40.1X1, Poisoning by heroin, accidental (unintentional)

T40.1X1A, Poisoning by heroin, accidental (unintentional initial encounter

T40.1X1D, Poisoning by heroin, accidental (unintentional subsequent encounter

T40.2X1, Poisoning by other opioids, accidental (unintentional)

T40.2X1A, Poisoning by other opioids, accidental (unintentional initial encounter

T40.2X1D, Poisoning by other opioids, accidental (unintentional subsequent encounter

T40.3X1, Poisoning by methadone, accidental (unintentional)

T40.3X1A, Poisoning by methadone, accidental (unintentional initial encounter

T40.3X1D, Poisoning by methadone, accidental (unintentional subsequent encounter

T40.3X1D, Poisoning by methadone, accidental (unintentional subsequent encounter

T40.4, Poisoning by, adverse effect of and underdosing of other synthetic narcotics

T40.491, Poisoning by other synthetic narcotics, accidental (unintentional)

T40.491A, Poisoning by other synthetic narcotics, accidental (unintentional initial encounter

T40.491D, Poisoning by other synthetic narcotics, accidental (unintentional subsequent encounter

T40.5, Poisoning by, adverse effect of and underdosing of cocaine

T40.5X1, Poisoning by cocaine, accidental (unintentional)

T40.5X1A, Poisoning by cocaine, accidental (unintentional initial encounter

T40.5X1D, Poisoning by cocaine, accidental (unintentional subsequent encounter

T40.5X2, Poisoning by cocaine, intentional self-harm

T40.5X2A, Poisoning by cocaine, intentional self-harm, initial encounter

T40.5X2A, Poisoning by cocaine, intentional self-harm, initial encounter

T40.5X2D, Poisoning by cocaine, intentional self-harm, subsequent encounter

T40.5X3, Poisoning by cocaine, assault

T40.5X3A, Poisoning by cocaine, assault, initial encounter

T40.5X3D, Poisoning by cocaine, assault, subsequent encounter

T40.5X4, Poisoning by cocaine, undetermined

T40.5X4A, Poisoning by cocaine, undetermined, initial encounter

T40.5X4D, Poisoning by cocaine, undetermined, subsequent encounter

T40.6, Poisoning by, adverse effect of and underdosing of other and unspecified narcotics

T43.62, Poisoning by, adverse effect of and underdosing of amphetamines

**Supplementary Document:** *Standard Operating Procedure for Chart Review of People Who Use Drugs*

Chart review instructions

**Encounters classified as PWUD if:**

- Documentation of drug use (current or former presence of + urine or serum toxicology, or presence of medications used for treatment of SUD in (1) ED note (2) Admission Note (3) Discharge
  - Drugs included: opiates, opioids, cocaine, amphetamines, other stimulants, inhalants, benzodiazepines, hallucinogens
  - People who use drugs, regardless of administration route

**Excluded if:**

- Alcohol, tobacco, nicotine, THC/cannabinoids were the ONLY drugs
- One-time attempted overdoses w/ intention of suicide as drug use
- Toxicology was positive following medically indicated use of substance (such as fentanyl for a medical procedure).
